# Roadmap to achieve 25% hypertension control in Africa by 2025

**DOI:** 10.5830/CVJA-2017-040

**Published:** 2017

**Authors:** Anastase Dzudie, Samuel Kingue, Anastase Dzudie, Karen Sliwa, Bongani Mayosi, Anastase Dzudie, Karen Sliwa, Brian Rayner, Dike Ojji, Aletta E Schutte, Marc Twagirumukiza, Albertino Damasceno, Seringe Abdou Ba, Abdoul Kane, Euloge Kramoh, Jean Baptiste Anzouan Kacou, Basden Onwubere, Ruth Cornick, Benedict Anisiuba, Ana Olga Mocumbi, Elijah Ogola, Mohamed Awad, George Nel, Harun Otieno, Ali Ibrahim Toure, Andre Pascal Kengne, Pablo Perel, Alm Adler, Neil Poulter

**Affiliations:** Department of Internal Medicine, Yaoundé Faculty of Medicine and Biomedical Sciences, Yaoundé, Cameroon; Department of Internal Medicine, Yaoundé Faculty of Medicine and Biomedical Sciences, Yaoundé, Cameroon; Department of Medicine, University of Cape Town, Cape Town, South Africa; Department of Medicine, University of Cape Town, Cape Town, South Africa; Department of Medicine, University of Cape Town, Cape Town, South Africa; Hatter Institute for Cardiovascular Research in Africa, Department of Medicine, Groote Schuur Hospital, University of Cape Town, Cape Town, South Africa; Hatter Institute for Cardiovascular Research in Africa, Department of Medicine, Groote Schuur Hospital, University of Cape Town, Cape Town, South Africa; Division of Nephrology and Hypertension, Department of Medicine, Groote Schuur Hospital and University of Cape Town, Cape Town, South Africa; Department of Medicine, Faculty of Health Sciences, University of Abuja; Cardiology Unit, Department of Medicine, University of Abuja Teaching Hospital, Gwagwalada, Abuja, Nigeria; Hypertension in Africa Research Team (HART); MRC Unit for Hypertension and Cardiovascular Disease, North-West University, Potchefstroom, South Africa; College of Medicine and Health Sciences, University of Rwanda, Kigali, Rwanda and African Society of Hypertension (AfSoH) Initiative, Heymans Institute of Pharmacology, Ghent University, Ghent, Belgium; Faculty of Medicine, Eduardo Mondlane University, Maputo, Mozambique; Le Dantec University Teaching Hospital, Dakar, Senegal; Service de cardiologie, Hôpital Général de Grand Yolf, Dakar, Senegal; Institut cardiologique d’Abidjan, Abidjan, Cote d’Ivoire; Institut cardiologique d’Abidjan, Abidjan, Cote d’Ivoire; Department of Medicine, University of Nigeria Teaching Hospital, Enugu, Nigeria; Knowledge Translation Unit, University of Cape Town Lung Institute and Department of Medicine, University of Cape Town, Cape Town, South Africa; Department of Medicine, University of Nigeria Teaching Hospital, Enugu, Nigeria; National Health Institute, Mozambique and University Eduardo Mondlane, Maputo, Mozambique; Department of Clinical Medicine and Therapeutics, University of Nairobi, Kenya; Division of Cardiology, University of Khartoum, Khartoum, Sudan; Pan-African Society of Cardiology (PASCAR), Cape Town, South Africa; Section of Cardiology, Department of Medicine, Aga Khan University Hospital, Nairobi, Kenya; Division of Cardiology, University of Khartoum, Sudan; Medical Research Council, Cape Town, South Africa; Science Advisory Unit, World Heart Federation, Geneva, Switzerland and London School of Hygiene and Tropical Medicine, London, UK; Science Advisory Unit, World Heart Federation, Geneva, Switzerland and London School of Hygiene and Tropical Medicine, London, UK; International Centre for Circulatory Health, Imperial College, London, UK

**Keywords:** hypertension,, roadmap, Africa, prevalence, control, blood pressure, action

## Abstract

**Background and aim:**

The Pan-African Society of Cardiology (PASCAR) has identified hypertension as the highest area of priority for action to reduce heart disease and stroke on the continent. The aim of this PASCAR roadmap on hypertension was to develop practical guidance on how to implement strategies that translate existing knowledge into effective action and improve detection, treatment and control of hypertension and cardiovascular health in sub-Saharan Africa (SSA) by the year 2025.

**Methods:**

Development of this roadmap started with the creation of a consortium of experts with leadership skills in hypertension. In 2014, experts in different fields, including physicians and non-physicians, were invited to join. Via faceto- face meetings and teleconferences, the consortium made a situation analysis, set a goal, identified roadblocks and solutions to the management of hypertension and customised the World Heart Federation roadmap to Africa.

**Results:**

Hypertension is a major crisis on the continent but very few randomised, controlled trials have been conducted on its management. Also, only 25.8% of the countries have developed or adopted guidelines for the management of hypertension. Other major roadblocks are either government and health-system related or healthcare professional or patient related. The PASCAR hypertension task force identified a 10-point action plan to be implemented by African ministries of health to achieve 25% control of hypertension in Africa by 2025.

**Conclusions:**

Hypertension affects millions of people in SSA and if left untreated, is a major cause of heart disease and stroke. Very few SSA countries have a clear hypertension policy. This PASCAR roadmap identifies practical and effective solutions that would improve detection, treatment and control of hypertension on the continent and could be implemented as is or adapted to specific national settings.

## Executive summary

The Word Health Organisation (WHO) estimated that the number of people affected by hypertension is highest in Africa, at about 46% of adults aged 25 years and older, compared to 35 to 40% elsewhere in the world. Many hypertensive Africans are unaware of their status, and are rarely treated or poorly controlled, making them at highest risk for stroke, and heart and renal disease.

African Union member states at the 2004 Addis Ababa meeting described hypertension as one of the continent’s greatest health challenges after HIV/AIDS. An urgency was recognised to develop and share best practices, including affordable and effective community-based programmes to screen and treat hypertension.

The WHO’s 2013–2020 global action plan calls upon the United Nations (UN) member states to take immediate action in preventing and controlling non-communicable diseases (NCDs). Target six of the action plan aims to achieve a 25% relative reduction in the prevalence of raised blood pressure or to contain this by 2020, according to national circumstances. State and government heads in the UN Political Declaration are committed to preventing and controlling NCDs through the establishment and strengthening of multi-sectoral national policies and plans.

The Pan-African Society of Cardiology (PASCAR) met several times to identify key actions for a hypertension roadmap on the continent. The PASCAR coalition identified several roadblocks hampering the control of hypertension on the continent, which exist at government/health-system, physician and patient levels and include the following.

## Government- and health system-related roadblocks

lack of established policies for controlling hypertension
poor political willingness to implement policies on NCDs
poor universal health insurance coverage, leading to out-ofpocket payment by most patients, which leads to poor access and adherence to treatment
lack of policies on antihypertensive medication procurement and distribution, resulting in stock shortages
lack of ad hoc screening and proper referral systems for patients identified at routine screening
inability of governments to effectively work with the private sector, non-governmental organisations (NGOs) and academia in a coordinated plan to tackle the burden of hypertension.

## Healthcare professional-related roadblocks

lack of appropriate evidence-based guidelines for healthcare professionals in individual countries
hypertension treatment guidelines are poorly implemented because of a lack of continuing medical education
a dearth of healthcare professionals (physicians, nurses and trained health workers) at primary care level with very low physician/patient ratio
lack of quality and affordable antihypertension medications.

## Patient-related roadblocks

poor awareness about hypertension and its consequences
poor adherence to drug therapy because of limited access to medication
difficulty in changing lifestyles, and false health beliefs that hypertension is curable, due to poor patient education.

## PASCAR 10-point action plan

The PASCAR hypertension task force identified a 10-point action plan, to be implemented by African ministries of health to achieve 25% control of hypertension in Africa by 2025.

1. All NCD national programmes should additionally contain a plan for the detection of hypertension.
2. Allocate appropriate funding and resources for the early detection, efficient treatment and control of hypertension.
3. Create or adopt simple and practical clinical evidence-based hypertension management guidelines.
4. Annually monitor and report the detection, treatment and control rates of hypertension, with a clear target of improvement by 2025, using the WHO STEPwise surveillance in all countries.
5. Integrate hypertension detection, treatment and control within existing health services, such as vertical programmes (e.g. HIV, TB).
6. Promote a task-sharing approach with adequately trained community health workers (shift-paradigm).
7. Ensure the availability of essential equipment and medicines for managing hypertension at all levels of care.
8. Provide universal access and coverage for detecting, treating and controlling hypertension.
9. Support high-quality research to produce evidence that will guide interventions.
10. Invest in population-level interventions for preventing hypertension, such as reducing high levels of salt intake and obesity, increasing fruit and vegetable intake and promoting physical activity.

African ministries of health, in their leadership roles, are called to adopt the 10-point action plan and customise it at a country level using a multi-sectoral approach. PASCAR calls on NGOs, all fraternal organisations, healthcare leaders and other members of the international community to join in this ambitious endeavour to support efforts by African ministries of health in reducing the burden of hypertension in Africa. Effective advocacy towards policy makers and politicians in national governments is particularly encouraged.

## Hypertension definitions

There is a graded relationship between blood pressure (BP) levels, as low as 115/75 mmHg, and cardiovascular disease (CVD) risk.^1^ However, hypertension is defined as the BP level above which treatments have been shown to reduce clinical events in randomised trials, which is accepted as ≥ 140 mmHg systolic and/or ≥ 90 mmHg diastolic BP. The classification of BP levels used for defining hypertension is presented in Table 1.

**Table 1:** Definitions of classes of raised blood pressure

*Category*	*SBP (mmHg)*	**	*DBP (mmHg)*
Optimal	< 120		< 80
Normal	120–129		80–84
High normal	130–139	or	85–89
Grade 1 hypertension (mild)	140–159	or	90–99
Grade 2 hypertension (moderate)	160–179	or	100–109
Grade 3 hypertension (severe)	≥ 180	or	≥ 110
Isolated systolic hypertension	≥ 140	and	< 90

SBP, systolic blood pressure; DBP, diastolic blood pressure

## Hypertension burden in Africa

Hypertension has progressively become a major threat to the well-being of people in sub-Saharan Africa (SSA). During the past four decades, the highest levels of BP worldwide have shifted from high-income countries (HIC) to low- and middle-income countries (LMIC) in South Asia and SSA.^2^ The WHO estimates that the prevalence of hypertension is highest in the African region, with about 46% of adults aged 25 years and older being hypertensive.^3^ This compares to 35% in the Americas and other HIC and 40% elsewhere in the world.^3^

High hypertension rates, ranging from 19.3% in Eritrea to 39.6% in the Seychelles, were reported for 20 African countries in WHO STEPS (STEPwise approach to surveillance) surveys conducted between 2003 and 2009.^4^ In a systematic review, the pooled prevalence in over 110 414 participants aged ± 40 years in 33 surveys was 30% (95% confidence interval: 27–34%).^5^ In Africa, the number of people with hypertension increased from 54.6 million in 1990 to 92.3 million in 2000, and 130.2 million in 2010. Under prevailing circumstances, this could increase to 216.8 million by 2030.^6^

## Gap in the care versus opportunity to control hypertension

**Fig. 1.**
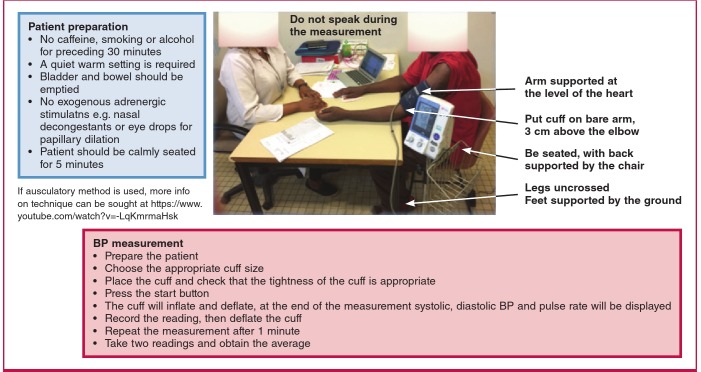
PASCAR recommendations for blood pressure measurement, thresholds and action required following appropriate office measurement.

The PASCAR task force recommends key steps for appropriate office measurement (Fig. 1). BP-lowering strategies that have shown their efficacy in HIC are likely to succeed in Africa. In Table 2, a synopsis is provided of currently published treatment guidelines differing regarding treatment thresholds.

**Table 2:** Blood pressure guidelines

**	*WHO PEN^2^*	*NICE 2011^2^*	*ESH/ESC 2013^3^*	*ASH/ISH 2014^4^*	*AHA/ACC/ CDC 2013^5^*	*US JNC 8 2014^6^*	*South Africa 2015^7^*	*Egypt 2013^8^*
Definition of hypertension (mmHg)	≥ 140/90	140/90 and daytime ABPM (or home BP) ≥ 135/85	≥ 140/90	≥ 140/90	≥ 140/90	Not addressed	≥ 140/90	≥ 140/90 (high risk) – 150/95 (low risk) and daytime ABPM (or home BP) ≥ 135/85
Drug therapy in low-risk patients after non-pharmacological treatment (mmHg)	> 160/100	≥ 160/100 or daytime ABPM ≥ 150/95	≥ 140/90	≥ 140/90	≥ 140/90	< 60 years, ≥ 140/90; ≥ 60 years, ≥ 150/90	≥ 140/90	≥ 140/90 for high risk and ≥ 160/100 for low risk
First-line therapy	< 55 years, lowdose thiazide diuretic and/or ACE inhibitor; ≥ 55 years, CCB and/or low-dose thiazide diuretic	< 55 years, ACE inhibitor or ARB; ≥ 55 years or African ancestry, CCB	ACE inhibitor or ARB; beta-blocker; CCB; diuretic	Low-dose diuretic		ACE inhibitor or ARB; CCB; diuretic CCB/diuretic in people of African ancestry	ACE inhibitor or ARB; CCB; diuretic CCB/diuretic in people of African ancestry	Any of diuretics, betablockers, CCB, ACEIs or ARBs. preferably a thiazide diuretic. In elderly (> 65 years) or in blacks, start with diuretic or CCB.
Beta-blockers as first-line drug	No	No (step 4)	Yes (in specific subgroups)	No (step 4)	No (step 3)	No (step 4)	No (step 4)	Yes, in specific e.g. young, particularly those with tachycardia
Diuretic	Thiazides,	Chlortalidone, indapamide	Thiazides, chlortalidone, indapamide	Thiazides, chlortalidone, indapamide	Thiazides	Thiazides, chlortalidone, indapamide	Thiazide or thiazide-like (indapamide)	Thiazides, chlorthalidone, amiloride or spironolactone
Initiate drug therapy with two drugs (mmHg)	Not mentioned	Not mentioned	In patients with markedly elevated BP or patients with high overall CV risk	≥ 160/100	≥ 160/100	≥ 160/100	≥ 160/100	Diuretic + beta-blockers/ CCB/ACEIs/ARBs if BP > 170/105
Blood pressure target (mmHg)	< 140/90	< 140/90; ≥ 80 years, < 150/90	< 140/90; elderly < 80 years, SBP 140–150, SBP < 140 in fit patients; elderly ≥ 80 years, SBP 140–150	< 140/90; ≥ 80 years, < 150/90	< 140/90; lower targets may be appropriate in some patients, including the elderly	< 60 years, < 140/90; ≥ 60 years < 150/90	< 140/90	150/95 in low-risk patients and in elderly (> 65 years). < 140/90: ≥ 2 risk factors, CKD, TOD < 130/80: HF or CKD when associated with proteinuria > 1 g/24 hours.
Blood pressure target in patients with diabetes mellitus (mmHg)	< 130/80	Not addressed	< 140/85	< 140/90	< 140/90; lower targets may be considered	< 60 years, < 140/90; ≥ 60 years, < 150/90	< 140/90	< 140/90 mmHg or < 130/80 if associated with proteinuria > 1 g/24 hours

ABPM, ambulatory blood pressure monitoring; ACC, American College of Cardiology; ACE inhibitor, angiotensin converting enzyme inhibitor; AHA, American Heart Association; ARB, angiotensin receptor blocker; ASH, American Society of Hypertension; BP, blood pressure; CCB, calcium channel blocker; CDC, Centers for Disease Control and Prevention; CKD, chronic kidney disease; CV, cardiovascular; ESC, European Society of Cardiology; ESH, European Society of Hypertension; ISH, International Society of Hypertension; NICE, National Institute for Health and Care Excellence; SBP, systolic blood pressure; TOD, target-organ damage; US JNC 8, Eighth US Joint National Committee; WHO PEN, World Health Organisation Package of Essential Non-communicable disease interventions.

^1^World Health Organisation. Implementation tools: package of essential non-communicable (PEN) disease interventions for primary healthcare in low-resource settings. Available at: http://apps.who.int/iris/bitstream/10665/133525/1/9789241506557_eng.pdf. Accessed April 8, 2015.

^2^National Institute for Health and Care Excellence. NICE guidelines [CG127]. Hypertension: clinical management of primary hypertension in adults. Available at: www.nice.org.uk/guidance/cg127/chapter/guidance. Accessed April 8, 2015.

^3^Mancia G, Fagard R, Narkiewicz K, et al. 2013 ESH/ESC guidelines for the management of arterial hypertension: the task force for the management of arterial hypertension of the European Society of Hypertension (ESH) and of the European Society of Cardiology (ESC). Eur Heart J 2013; 34: 2159–2169.

^4^Weber MA, Schiffrin EL, White WB, et al. Clinical practice guidelines for the management of hypertension in the community a statement by the American Society of Hypertension and the International Society of Hypertension. J Hypertens 2014; 32: 3–15.

^5^Go AS, Bauman MA, Coleman King SM, et al. An effective approach to high blood pressure control: a science advisory from the American Heart Association, the American College of Cardiology, and the Centers for Disease Control and Prevention. J Am Coll Cardiol 2014; 63: 1230–1238.

^6^James PA, Oparil S, Carter BL, et al. 2014 evidence-based guideline for the management of high blood pressure in adults: report from the panel members appointed to the Eighth Joint National Committee (JNC 8). J Am Med Assoc 2014; 311: 507–520.

^7^Seedat Y, Rayner B, Veriava Y. South African hypertension practice guideline 2014. Cardiovasc J Afr 2014; 25(6): 288–194.

^8^The Egyptian Hypertension Society: Egyptian hypertension guidelines. Egypt Heart J 2014; 66(2): 79–132.

**Fig. 2.**
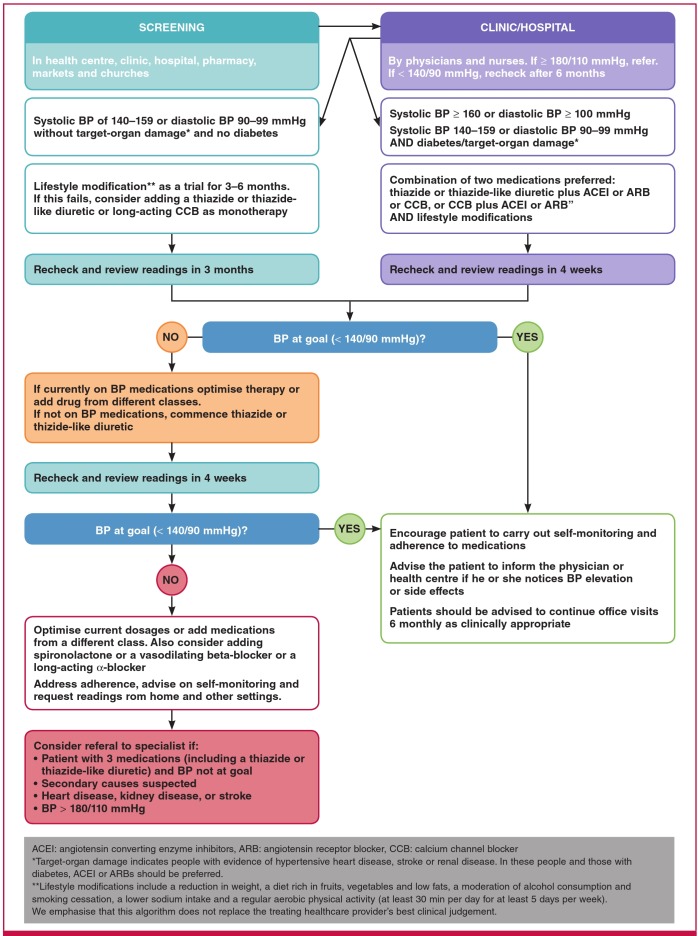
PASCAR hypertension treatment algorithm

A simple and practical treatment algorithm using these thresholds is recommended (Fig. 2). Our schedule should consider patient costs (including transport and loss of wages because of time off to attend clinic visits), which affect treatment adherence and burden to the healthcare system.

Because of the asymptomatic nature of hypertension, longterm medication adherence is poor. Patients and healthcare practitioners must be educated on non-pharmacological BP control methods (see Fig. 2). We encourage patient education using text messages, e-mails or social media (WhatsApp or Facebook), all of which are progressively available and affordable in Africa. We also encourage face-to-face education by traditional and religious leaders.

The discrepancy between best practice (based on highquality evidence) and the care provided in routine clinical practice is called the ‘care gap’. This includes situations in which interventions with proven efficacy are under-utilised. This description is most marked in Africa, where it is favoured by poverty and inadequate utilisation of existing resources. Despite strong evidence of the management benefit, this can reflect as poor awareness and control of CVD rates or risk factors.

**Fig. 3.**
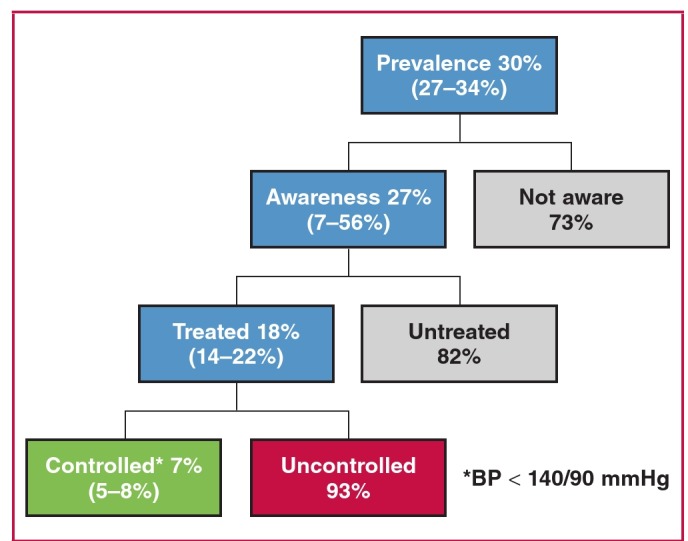
Prevalence, awareness, treatment and control of hypertension in Africa. Numbers are from Ataklte et al. Burden of undiagnosed hypertension in sub-Saharan Africa: A systematic review and meta-analysis.^5^

Current data on awareness and hypertension control rates in SSA are from a wide range of studies differing in methodology and limiting the opportunity for reliable comparisons. However, available data show that the high prevalence of hypertension in Africa, as in other LMICs, is coupled with low awareness and control rates (Fig. 3), a reflection of a maximal care gap.^5^-^8^

In 2011, UN member states acknowledged at the highest international level that premature deaths from NCDs reduce productivity and curtail economic growth, causing significant social challenges in most countries.9 In 2015, the previous target of 25% reduction in rate of premature mortality from NCDs by 2025 was extended to a reduction of 33% by 2030, through prevention, treatment and promotion of mental health and wellbeing.^10^

Since 2004, the African Union, in a pro-active approach, named hypertension one of the continent’s greatest health challenges after HIV/AIDS. After more than a decade, this political enthusiasm, which is crucial for the development and implementation of any healthcare policy, has yet to be translated into public health action. The role of hypertension experts is key to facilitate the states’ action to adequately contain this threat. In a simple and practical hypertension policy, the PASCAR approach emphasises working in collaboration with all other stakeholders to set clear goals and define priority actions and minimum standards of African healthcare systems.

## The World Heart Federation roadmap and other relevant initiatives

As part of the WHO’s target in reducing heart attacks and stroke by 2025, the World Heart Federation (WHF) launched a roadmap focusing on raised BP during the 2015 World Health Assembly in Geneva.^11^ Herein the routes are described towards reducing premature cardiovascular mortality rate by 25%,^11^ focusing on presenting practical steps for hypertension control. For effective hypertension control, four population groups were identified: people who are unaware of their BP status; those who are aware of having raised BP but it is uncontrolled; those who are aware of their raised BP, which is under control; and those who are aware of having normal BP.

After identifying the target population, practical steps are provided for improving hypertension management. These include opportunistic screening for awareness of BP status and effective drug treatment for high BP.

The use of generic antihypertensive medications rather than proprietary medications is encouraged, to substantially reduce the cost of care, but with a caveat for the need to ensure quality generic medications. Bearing in mind the holistic nature of healthcare delivery, this initiative identified health-system requirements to achieve BP management targets and include human, physical and intellectual resources, healthcare delivery, healthcare recipients, financing, and governance and information systems.

With global information technology tools available, the suggestion of using e-health, particularly m-health, for patient education is a very feasible approach in the guidelines. If well applied, this could be a useful tool in hypertension control. This roadmap is anticipated to substantially bridge the gap between HIC and LMIC in terms of hypertension management and control policy. The WHO Package of Essential Non-communicable (PEN) Disease Interventions for Primary Healthcare in Low-Resource Settings is an integrated approach to NCDs focusing exclusively on primary healthcare in low-resource settings.^12^

The WHF roadmap provides a global framework to reduce CVD mortality, focusing on evidence-based interventions. Strong emphasis is placed on health systems, cost-effectiveness and subsequent evaluation of programmes. Hypertension as a single risk factor, and an entry point to prevent CVD rather than the absolute-risk approach, provides a framework to identify roadblocks in implementing evidence-based interventions. Hypertension seldom occurs in isolation, co-existing with other CVD risk factors, contributing to the absolute-risk status.

The PASCAR roadmap strongly emphasises hypertension as a global health crisis and major threat. We hope that hypertension screening will increase in the next eight years, resulting in a paradoxical increase in the prevalence of hypertension. For this reason, the task force’s target is to increase treatment and control rates among the treated subjects by 25% in the SSA region by 2025.

We identified roadblocks to the control of hypertension in the African region and proposed solutions to these roadblocks, thus defining the best strategy to achieve this in SSA. Because the epidemic of NCDs is driven by globalisation, urbanisation, demographic trends and socio-economic conditions,^13^ interventions to reach our targets are required from the health sector and other governmental sectors, along with civil society and the private sector.

Therefore, guidance is provided for policy makers, healthcare professionals (nurses, general practitioners, family doctors, internists, cardiologists, nephrologists and other hypertension specialists), patients, the private sector and the public, including civil society, on controlling hypertension to reduce premature mortality from CVD. To guide the action of stakeholders, we also highlight the importance of reaching minimum standards (Table 3) for the health systems of countries to achieve the 25% hypertension control target. Implementation of these solutions and suggestions on customising the overall strategy at a country level are discussed.

**Table 3:** Minimum care for hypertension management at each healthcare level in Africa

*Basic staff, equipment, test and medication*	*Level of care*		
**	*Primary*	*Secondary*	*Tertiary*
**	*Trained health worker or nurse*	*Medical Practitioner*	*Specialist*
Basic equipment			
Automated blood pressure devices, or calibrated sphygmomanometer, either mercury or oscillometric plus appropriate cuffs	+++	+++	+++
Home blood pressure devices		+	+++
Ambulatory blood pressure devices		+/–	+++
Tape measure for waist circumference	+++	+++	+++
Scale for weight	+++	+++	+++
Stadiometer for height	+++	+++	+++
Standard 12-lead ECG		++	+++
Glucometer	+	+++	+++
Funduscope		++	+++
Stethoscope	+++	+++	+++
Basic tests			
Urine dipsticks for protein, blood and glucose	+++	+++	+++
Standard 12-lead ECG recording		++	+++
Glucometer strips for testing glucose	+	+++	+++
Na+, K+ and creatinine with calculation of eGFR	+	++	+++
Cholesterol		+	+++
Glycated haemoglobin (HbA_1c_)	+	++	+++
Chest radiograph		+/–	+++
Basic medication classes with examples*			
Thiazide or thiazide-like diuretic (hydrochlorothiazide, indapamide, chlorthalidone)	+++	+++	+++
Calcium channel blockers (amlodipine, nicardipine, long-acting nifedipine)	+++	+++	+++
Angiotensin converting enzyme inhibitor (enalapril, lisinopril, perindopril, ramipril)	+	+++	+++
Angiotensin receptor blockers (candesartan, valsartan, losartan)		+++	+++
Vasodilating beta-blockers (nebivolol, bisoprolol, carvedilol)		+++	+++
Spironolactone		+++	+++
Long-acting α-blocker (doxazocin)		+	+
Combinations of blood pressure-lowering medications	+	+++	+++

+++: strongly recommended; ++ moderately recommended, +: recommended; –: not done; +/–: done if facilities are available.

*Availability of drugs at each level of care has been indicated and recommended here for initiation only, all drugs can be used once initiated by a medical practitioner.

A trained healthcare worker may initiate and follow up some medication.

The WHF roadmap provides a general framework that could be useful for LMICs, however, to be implemented it should be customised according to the local context. With PASCAR’s leadership and the contribution of other professional organisations, this approach seems to be at the right time to turn the many hypertension challenges in Africa into immense opportunities. Although population-based strategies for lowering BP may be cost-effective, they are not the focus of this roadmap, but we recognise these would be beneficial.

## Methods

In January 2014, panel members who were appointed to develop the PASCAR roadmap were invited to join the PASCAR task force on hypertension. Based on their expertise and leadership in hypertension, 41 nominees from 21 countries received invitations, with 95% responding positively. These experts included cardiologists, nephrologists, public health physicians, researchers (including clinical trialists), nurses, pharmacologists, evidence-based medicine specialists and guideline developers.

During the first face-to-face meeting held in Nairobi on 27 October 2014,^14^ the group acknowledged the lack of a continental strategy to address the hypertension crisis. A decision was taken to develop a roadmap for the prevention and management of hypertension in Africa as a matter of urgency under the auspices of the WHF.

To customise the WHF BP roadmap for Africa, the core group performed a comprehensive literature search and communicated with the WHF from November 2014 to July 2015 via teleconference and e-mail. After receiving and comprehending the WHF roadmap document, task force members held a second face-to-face meeting in London on 30 August 2015, to make suggestions on its relevance and customisation. A detailed presentation of this roadmap was reviewed and discussed by PASCAR task force members, hypertension experts and leaders of hypertension societies via e-mail, with WHF feedback.

Development of a warehouse for African guidelines and clinical trials on hypertension was also reviewed. Finally, the steps in developing the African roadmap for reducing CVD mortality rates through BP control was planned.

The first draft of the PASCAR roadmap for hypertension management and control was presented in Mauritius on 4 October 2015. Attendees were 13 presidents of national cardiac societies or representatives, the president of the International Forum for Hypertension Control and Cardiovascular Disease Prevention in Africa and representative of the International Society of Hypertension, a representative of the African Heart Network, members of the PASCAR task force on hypertension, and scientists from the WHF. The draft was reviewed and oral and e-mail comments were received from participants. The WHO PEN programme^12^ was compared with the PASCAR hypertension roadmap to ensure complementarity between the two documents.

The second version of the roadmap draft was submitted to a core group for internal review from October to December 2015. In March 2016, a selected group of hypertension experts from 12 French-speaking countries met in Yaoundé to discuss the algorithm and the draft.^15^ Comments were received and the draft was amended.

The task force reviewed the final draft of the roadmap in Mexico in June 2016, which was then submitted for external peer-review by three independent experts in hypertension and policy development. The subsequent review was done by a group of experts in cardiology, nephrology, primary care and research (including clinical trials). Comments were reviewed and discussed by the panel and incorporated into a revised and final document.

## PASCAR searches and surveys on the status of hypertension policy programmes and clinical practice guidelines

From May to July 2015, an internal PASCAR survey was conducted, aiming to determine which African countries ran hypertension control programmes focusing on policy. Using the Survey Monkey software tool,^16^ national hypertension experts from 40 countries were asked whether a hypertension policy programme was operating in their country and could be judged as being ‘dormant’, ‘not much active’, ‘active’, or ‘very much active’.

Among the responders (n = 127) representing 27 SSA countries, we noticed that up to 63.7% did not have a hypertension policy programme or that it was dormant or not very active. This regrettable situation highlights the importance of a continental initiative to develop a hypertension policy to address BP control from a population-wide and high-risk approach.

Evidence has shown that explicit clinical practice guidelines (CPGs) do improve the care gap by providing practitioners and health-service users with synthesised quality evidence regarding decision-making.^17^ In another PASCAR study, we assessed the existence, development and use of national guidelines for the detection and management of hypertension in the African region, regardless of quality.

Between May and July 2015, CPGs for hypertension were searched, using a scientifically developed search strategy. Searches were done using Google and PubMed. Search terms included (country name) AND (hypertension OR HTN OR high blood pressure) AND (clinical practice guidelines OR treatment guide). French, Portuguese and Spanish translations were included in the search strategy.

Websites of ministries of health, national medical associations and the WHO were hand-searched, authors were e-mailed, and requests were sent on Afronets to obtain copies of CPGs for hypertension. To be included in the search, the CPGs had to be available and provided in full-text versions for assessment by the review team, comprising three independent authors. CPGs from Europe or South America or those that could not be obtained were considered non-existent. Two national hypertension experts were contacted for confirmation on countries for which we could not find CPGs on hypertension. CPGs published in peerreviewed journals needed to be readily accessed by end-users. E-mail messages were used for further clarification.

**Fig. 4.**
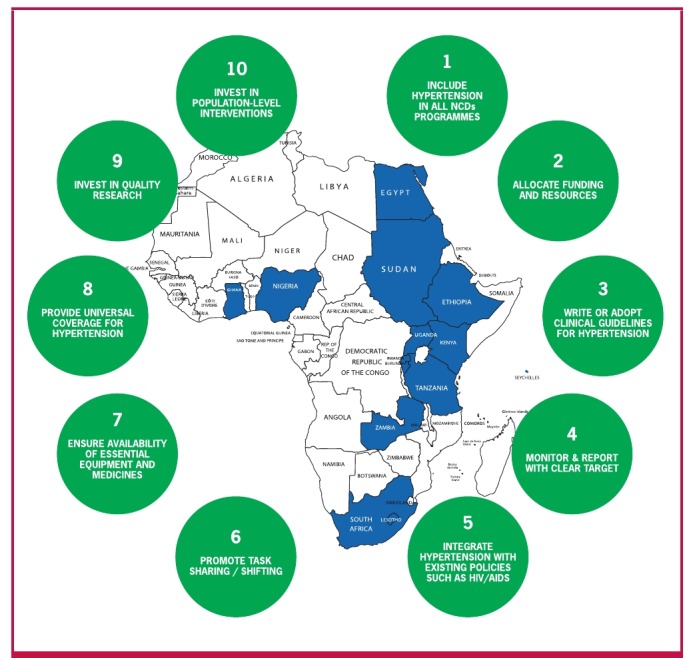
2015 map of African countries with evidence of existing clinical practice guidelines for hypertension management and 10 actions to reduce the hypertension burden in Africa

In Fig. 4, the 2015 map is presented of countries with clear evidence of the existence of national guidelines for detection and management of BP across Africa. Only 16 (25.8%) out of 62 countries had CPGs complying with our search criteria. No evidence of CPGs on hypertension management could be found for the other 46 (74.2%) countries. Given that the only existing multinational expert recommendations for the management of hypertension in Africa dates back to 2003 and has not been updated since,^18^ we concluded that there is a legitimate, pressing need to support African ministries of health with a clear hypertension roadmap.

## PASCAR roadmap to decrease the burden of hypertension in Africa

To reduce the incidence of CVD through treating hypertension in the African region, it will be necessary to increase the rates of detection, treatment and control of the disease. The 10 actions that need to be undertaken by African ministries of health to achieve a 25% control of hypertension in Africa by 2025 (Fig. 4) are listed below and we include an explanation as to why (bullets) and how (dashes) this needs to be done.

1. All NCD national programmes should additionally contain a plan for the detection of hypertension.
  • The hypertension crisis has yet to receive an appropriate response in SSA.^19^
  • Incidence of hypertension increased by 67% since 1990 and was estimated to cause more than 500 000 deaths and 10 million years of life lost in 2010 in SSA.^20^,^21^
  • Hypertension is the main cause of stroke, heart failure and renal disease in SSA.
  • Stroke, which is a major complication of uncontrolled hypertension, has increased to 46% since 1990 and essentially affects breadwinners.^20^
  • Failure to control hypertension and its economic repercussions through revising health policies and services endangers the economic prosperity of all African nations.^22^
  – All SSA countries should have adopted and should follow the WHO global agenda of reducing NCDs by 2020.
  - When reporting to the Ministry of Health and the WHO, stakeholders should report specifically on hypertension.
  - National cardiac and hypertension societies should monitor the prevalence, awareness and control rates of hypertension and report to PASCAR.
  - Government, private sector, academia and community organisations should pay attention to this report and work together for a reduction in hypertension prevalence.
2. Allocate appropriate funding and resources for the early detection, efficient treatment and control of hypertension.
  • The costs of priority interventions for NCDs, including hypertension, have been shown to be small and countries are receiving global funds.
  • No new global funding is needed to implement the 10 actions for controlling hypertension.
  • Comprehensive implementation to control hypertension and reduce salt intake is affordable in all countries.
  • The current increasing burden of uncontrolled hypertension is a barrier to the development of all African nations.
  • Funding to support civil society and health organisations will contribute to developing and implementing appropriate health policies to control hypertension.
  • Funding is needed to support dissemination of best practices to detect, manage and control NCDs within Africa.
    –– Increase healthcare budgets in Africa to align with the WHO global action plan of 2013–2020, which has already been adopted by all SSA countries.
    –– Realign existing funding with the emerging hypertension threat that SSA populations are experiencing.
    –– Dedicate a clear percentage of the health budget to hypertension policy.
    –– Use existing resources more efficiently.
    –– Develop innovative funding mechanisms, including additional alcohol and tobacco taxes.
    –– National cardiac and hypertension societies should monitor the hypertension/NCD-related budget every two years and advocate otherwise for improvement.
3. Create or adopt simple and practical clinical evidence-based hypertension management guidelines.
  • The role of simple and practical guidelines is crucial for managing NCDs at large, and hypertension specifically.
  • In 2015, only 25% of SSA countries had developed or adopted clinical guidelines for managing hypertension (Fig. 4).
  • New scientific knowledge guides implementation and efficiency in developing guidelines according to the best actual practices.
    –– PASCAR will develop and regularly update continental guidelines with a simple care algorithm (Fig. 2) for detecting, treating and controlling hypertension. National cardiac societies are called upon to adopt or adapt to the country’s circumstances where appropriate.
    –– Alternatively, the WHO HEARTS technical package for CVD management in primary healthcare overtakes WHO PEN12 and provides a comprehensive CVD control approach,^23^ with the possibility of integrating hypertension as a risk factor.
    –– PASCAR has defined and will regularly update the minimum standards (Table 3) to control hypertension, which need to be achieved by each SSA country. Countries are called upon to adopt and implement these.
4. Annually monitor and report the detection, treatment and control rates of hypertension, with a clear target of improvement by 2025, using the WHO STEPwise surveillance in all countries.
  • The success of all NCD interventions, including hypertension policy, will depend on how specific, measurable, achievable, realistic and time-bound the objectives are.
  • A framework for national and continental monitoring, reporting and accountability will ensure that the returns on investments in hypertension and other NCDs meet the expectations of all partners.
    –– The WHO STEPwise approach to NCD risk-factor surveillance should be strengthened in all African countries to report on detecting, treating and controlling hypertension annually.
    –– BP to be measured at all relevant clinical encounters.
    –– Regular representative population surveys are effective in monitoring trends of key risk factors and the uptake of priority interventions, such as the WHO STEPS approach to monitor NCD risk factors.
    –– National cardiac and/or hypertension societies should measure the level of coverage for some sentinel sites (communities, industries, primary healthcare centres, etc.) and report to PASCAR.
    –– National cardiac and/or hypertension societies should take responsibility for reporting progress in hypertension control, mobilising resources, developing policy and identifying best practices.
    –– The monitoring and reporting team in sentinel sites will ensure that people know their BP, hypertensives receive appropriate treatment, BP is controlled and they remain on treatment.
5. Integrate hypertension detection, treatment and control within existing health services, such as vertical programmes (e.g. HIV, TB).
  What the medical community learned from the large-scale management of TB and HIV/AIDS should be successful in managing hypertension.
    –– The government, private sector, academia and community organisations should work together to align plans for detecting, treating and controlling hypertension with other ongoing programmes.
    –– Emphasis should be placed on (1) standardised treatment protocols, (2) identification and availability of affordable and effective drugs, and (3) service delivery, as with TB and HIV programmes.
6. Promote a task-sharing approach with adequately trained community health workers (shift-paradigm).
  • SSA carries 11% of the world population, 25% of the global burden of disease, with only 3% of the world’s health labour force, and has a global health expenditure of less than 1%.^24^
  • These health-worker shortages are a major barrier to controlling hypertension in Africa.
  • Clear evidence exists that health staff without formal professional training can be adequately trained to effectively detect people with severe hypertension.^25^
  • Using trained community health workers (CHW) to detect hypertension would free health professionals in Africa to treat and control the condition.
  • Well-trained nurses, general and family physicians can adequately manage uncomplicated hypertension, freeing specialists for more severe cases.
    –– Design a course to train CHW in detecting hypertension, providing information and educating the community.
    –– Train 250 000 CHW to detect hypertension by 2025.
    –– Design special courses reinforcing health staff capacity to manage hypertension.
    –– Use an online system to train at least 50 000 certified nurses and 25 000 certified general physicians to take appropriate decisions regarding detecting, treating and controlling hypertension by 2025.
    –– PASCAR and national cardiac societies will design the course, and national recertification may be required after training.
7. Ensure the availability of essential equipment and medicines for managing hypertension at all levels of care.
  • Target 8 of the global action plan acknowledges the need to improve the availability of affordable BP machines and medicines for the poor.^26^
  • Target 9 of the global action plan is an 80% availability of affordable basic technologies and essential medicines, including generics, required to treat major NCDs in public and private facilities.^26^
  • Access to affordable and good-quality drugs for hypertension is important for all LMICs, and especially SSA.^26^
    –– Governments and societies should be willing to prioritise hypertension control and provide low-cost BP machines and medications.
    –– PASCAR and national cardiac and hypertension societies have adopted a hypertension treatment algorithm, suggesting the use of high-quality antihypertensive medications (Fig. 2).
    –– The ongoing randomised clinical trial, Comparing Three Combination Therapies in Lowering Blood Pressure in Black Africans (Creole), will provide more evidence-based information on the most efficacious of three ‘free’ combinations of two antihypertensive agents on 24-hour ambulatory systolic BP.^27^
    –– PASCAR has defined minimum standards for BP machines and drug availability and affordability to control hypertension in Africa (Table 3).
    –– PASCAR and national cardiac and hypertension societies should strongly advocate making antihypertensive medications available and more affordable to patients.
    –– Governments should encourage adding to and periodically updating the hypertension medications on their national essential medicine list.
    –– Governments should subsidise the cost of and remove import duties on these essential medications.
    –– Governments should put in place an efficient, highquality monitoring process of medicines.
    –– Donor organisations and pharmaceuticals should be engaged in making these medications affordable.
    –– PASCAR will regularly measure the proportion of the population with access to affordable, essential drugs in sentinel sites.
8. Provide universal access and coverage for detecting, treating and controlling hypertension.
  • There are proven cost-effective lifestyle and medical interventions to prevent and manage hypertension. However, in Africa, uptake is still unacceptably low.^3^
  • Universal health coverage will be the main step forward to ensure that persons with hypertension have access to effective, affordable and accessible care.
    –– Governments must have the political will to acknowledge the hypertension crisis, and the commitment to convince their parliaments to approve budgets needed for universal coverage.
    –– Failure to implement universal coverage may result in increased healthcare expenditure on the complications of hypertension.
9. Support high-quality research to produce the evidence that will guide interventions.
  • Data from randomised, controlled trials on hypertension management are lacking in SSA.
  • Research is vital in formulating a sound healthcare policy to evaluate the performance of interventions in hypertension control and take managerial decisions in the overall NCD policy.^28^
  • Research into hypertension in Africa should be essential, especially where it can inform resource-allocation decisions.
    –– African governments should encourage all multidisciplinary, multidirectional and collaborative approaches at national and international levels, and take a firm commitment to develop research guided through priority intervention, as suggested by the WHO.^26^
    –– National cardiac and/or hypertension societies should take responsibility for identifying research priorities, building national and international research networks and partnerships, and advocating for investment in research to support best practices.
    –– PASCAR, with its good continental research network, will continue taking the leadership for research training and funding while ensuring to develop and sustain research activities to guide cost-effective interventions for hypertension control.
10. Invest in population-level interventions for preventing hypertension, such as reducing salt intake and obesity levels, increasing fruit and vegetable intake and promoting physical activity.
  • The relationship between BP and the risk of developing stroke or heart disease is ongoing, starting at a systolic pressure > 115 mmHg.^1^
  • Hypertension is a preventable cause of morbidity and mortality.
  • High-quality evidence in non-acutely ill adults shows that reduced sodium intake reduces BP.^29^
  • These two previous facts highlight the importance of highrisk and population-based strategies in BP management and control.
    –– Therefore, advocate for a healthy public policy and large national programme for hypertension prevention and control.
    –– Use national multi-sectoral policies and plans that specifically address physical activity and nutrition, including dietary salt, in preventing hypertension and NCDs.
    –– Wider implementation of successful governmental actions including smoke-free policies, marketing of unhealthy foods and alcohol, sin taxes (e.g. sugar taxes), and regulation of sodium content in processed foods.

## How to adapt the PASCAR 10 actions at country level

This roadmap can be implemented as is or adapted to overcome local barriers and develop solutions that are more relevant to specific national settings. In the latter case, we recommend that national roadmaps be developed, using a multi-sectoral approach in collaboration with inter-governmental organisations, heart health advocacy foundations, cardiovascular scientific organisations, healthcare leaders, providers from primary and specialised care, private-sector stakeholders and people affected by CVD.

Effective advocacy towards policy-makers and politicians in national governments is mandatory for success. Screening among politicians might be an effective way to increase awareness and encourage governments to act.

The PASCAR task force recommended the following steps:

• Step 1: where applicable, national cardiac societies (otherwise national hypertension societies or cardiovascular specialists) should take the leadership to develop and convene a multisectoral coalition against hypertension. At this step, persuading the government and all other stakeholders to collaborate is essential.
• Step 2: this coalition will then assess the epidemiological profile of hypertension and review and synthesise existing official data and published and unpublished literature. This step also includes a map of all existing policies.
• Step 3: the coalition conducts policy dialogues with multiple local stakeholders. Local problems, specific barriers to hypertension control and potential solutions should be discussed and appropriate strategies selected according to context. At this step, it is important to understand existing policies and their current effect. Within the same nation, appropriate strategies may also need adaptation. Some stakeholders who will be invited to the policy dialogue include the ministry of health, various health sector staff (physicians and non-physicians), health workers, key opinion leaders such as politicians and religious people, and also alternative medicine specialists and traditional healers, who may have a significant influence on people with hypertension in some settings.
• Step 4: the coalition develops a clear national strategy and time-bound plan for detecting, treating and controlling hypertension.

The PASCAR coalition against hypertension takes responsibility for fostering the development of national roadmaps and supporting national cardiac and hypertension societies at all levels.

## Conclusions

Although there is significant scientific evidence that costeffective lifestyle and medical interventions could control hypertension and prevent health-threatening complications, such as heart disease and stroke, the African region still bears a very high disease prevalence, coupled with poor rates of detection, treatment and control. This context is a barrier to the achievement of the universal global action plan and gives reasons for urgent action.

The PASCAR task force on hypertension roadmap was conceived by a variety of leaders and stakeholders in the field to provide the most appropriate strategy to have 25% control of hypertension by 2025. The roadmap identifies major barriers to disease control and priority areas of intervention, and 10 actions to improve the control of hypertension by 2025 are proposed. The most important steps to put forth in this continental roadmap include:

1. Advocate for government leadership and policy.
2. Allocate funding and resources.
3. Design simple and practical guidelines.
4. Promote large-scale screening.
5. Integrate hypertension detection, treatment and control in all existing programmes.
6. Promote task sharing and expand the scope of practice.
7. Promote the use of inexpensive, good-quality BP machines and generic medications.
8. Promote universal coverage for hypertension diagnosis and management.
9. Support high-quality research to produce the best evidence for interventions.
10. Invest in population preventive measures.

This is a unique moment in history for the African CVD community to have worked with global leaders in the field in defining a clear agenda to address the hypertension crisis. Support for this programme from the African Union and all stakeholders will help achieve the WHO global action plan of 2013–2020 for NCD reduction, specifically focusing on heart attack, stroke and other CVDs. The WHO and other UN organisations will support national efforts with upstream policy advice and sophisticated technical assistance, ranging from assisting governments to setting national targets in implementing relatively simple steps, which can make a huge difference.
